# Precise film dosimetry for stereotactic radiosurgery and stereotactic body radiotherapy quality assurance using Gafchromic™ EBT3 films

**DOI:** 10.1186/s13014-016-0709-4

**Published:** 2016-10-04

**Authors:** Ning Wen, Siming Lu, Jinkoo Kim, Yujiao Qin, Yimei Huang, Bo Zhao, Chang Liu, Indrin J. Chetty

**Affiliations:** Department of Radiation Oncology, Henry Ford Health Systems, 2799 West Grand Blvd, Detroit, MI USA

**Keywords:** Stereotactic radiosurgery, Stereotactic body radiation therapy, Gafchromic films, Quality assurance

## Abstract

**Purpose:**

The purpose of this study is to evaluate the dosimetric uncertainty associated with Gafchromic™ (EBT3) films and establish a practical and efficient film dosimetry protocol for Stereotactic Radiosurgery (SRS) and Stereotactic Body Radiotherapy (SBRT).

**Method and materials:**

EBT3 films were irradiated at each of seven different dose levels between 1 and 15 Gy with open fields and standard deviations of dose maps were calculated at each color channel for evaluation. A scanner non-uniform response correction map was built by registering and comparing film doses to the reference ion chamber array-based dose map delivered with the same doses. To determine the temporal dependence of EBT3 films, the average correction factors of different dose levels as a function of time were evaluated up to 4 days after irradiation.

An integrated film dosimetry protocol was developed for dose calibration, calibration curve fitting, dose mapping, and profile/gamma analysis. Patient specific quality assurance (PSQA) was performed for 83 SRS/SBRT treatment plans, and analysis of the measurements and calculations are presented here.

**Results:**

The scanner response varied within 1 % for the field sizes less than 5 × 5 cm^2^, and up to 5 % for the field sizes of 10 × 10 cm^2^ for all color channels. The scanner correction method was able to remove visually evident, irregular detector responses for larger field sizes. The dose response of the film changed rapidly (~10 %) in the first two hours and became smooth plateaued afterwards, ~3 % change between 2 and 24 h. The uncertainties were approximately 1.5, 1.7 and 4.8 % over the dose range of 3~15 Gy for the red, green and blue channels. The green channel showed very high sensitivity and low uncertainty in the dose range between 10 and 15 Gy, which is suitable for SRS/SBRT commissioning and PSQA. The difference between the calculated dose and measured dose of ion chamber measurement at isocenter was −0.64 ± 2.02 for all plans, corresponding to a 95 % confidence interval of (−1.09, −0.26). The percentage of points passing the 3 %/1 mm gamma criteria in absolute dose, averaged over all tests was 95.0 ± 4.2.

**Conclusion:**

We have developed the EBT3 films based dosimetry protocol to obtain absolute dose values. The overall uncertainty has been established to be 1.5 % for SRS and SBRT PSQA.

## Introduction

Flattening Filter Free (FFF) modalities have been widely used for Stereotactic Radiosurgery (SRS) and Stereotactic Body Radiotherapy (SBRT) procedures, because of the high dose rate, sharp penumbra, reduced leaf transmission and head scatter associated with FFF beams [1–3]. Modern radiation therapy modalities, including intensity modulated radiation therapy (IMRT) and volumetric modulated arc therapy (VMAT), produce SRS/SBRT plans with highly irregular and steep dose gradient distributions. IMRT/VMAT treatment beams are often heterogeneous and complex, consisting of many small beam apertures realized by a multi-leaf collimator (MLC). Accurate verification of such complex treatment fields is challenging. The conventional method for patient specific quality assurance (PSQA) of measuring a point dose using an ionization chamber (IC) or fluence using a two-dimensional (2D) array is inadequate for highly modulated treatment fields with sharp dose gradient fall off [4–6].

Gafchromic™ EBT3 film has been introduced to eliminate measurement orientation effects as well as Newton rings formed during film scanning [7]. EBT3 Film dosimetry has been used most commonly for relative dose analysis [8]. The absolute dose map is built by normalizing a selected reference point to the reading of a dosimeter, which is inadequate for SRS and SBRT PSQA considering the volume size of the dosimeters in general. There has been no study to evaluate the characteristics of EBT3 Gafchromic™ films on FFF beams and high dose range in the SRS/SBRT dose regimen. This study presents a practical and efficient film dosimetry protocol for SRS and SBRT PSQA using FFF photon beams. Uncertainties associated with the film channels coupled with a flatbed scanner were evaluated in the absolute dose analysis.

## Materials and methods

### Film scanning

**Fig. 1:**
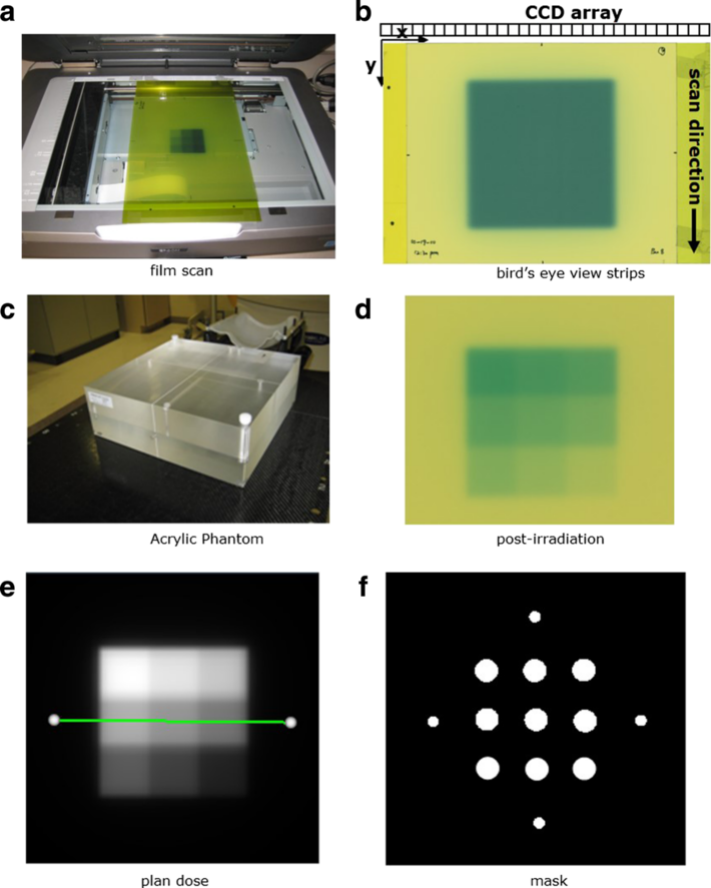
**a** film scanning orientation with the longer side of film aligned to the scanner detector array. **b** A bird’s eye view of film scanning orientation with film strips attached for positioning accuracy. **c** Acrylic phantom for film dose calibration. **d** A calibration film irradiated with a nine 2 × 2 cm^2^ square dose pattern. **e** The corresponding planar isodose distribution. **f** A mask pattern used to sample optical density values to establish the calibration curve

An Epson Expression 10000XL document flat-bed scanner (Seiko Epson Corp, Nagano, Japan) was used to scan the films. Each film was scanned in the center of the scanner bed to allow for better scanner response uniformity [9] and in the landscape orientation with the longer side of film parallel to the scanner detector array (Fig. 1a) [10, 11]. To improve film placement reproducibility and reduce orientation-dependent response variations, two film strips were attached to the scanner bed and the shorter film side was aligned against the strip (Fig. 1b) [12]. The film strips were used instead of an opaque frame in order to provide similar light scattering characteristics near the film borders. The films were scanned in transmission mode for better scanning stability [13, 14] with settings of 150 dot per inch and 48 bit RGB mode (16 bits per color channel). Images were exported in tagged image file format (TIFF) for analysis and image processing filters were disabled.

### Scanner Non-uniform response correction

**Fig. 2:**
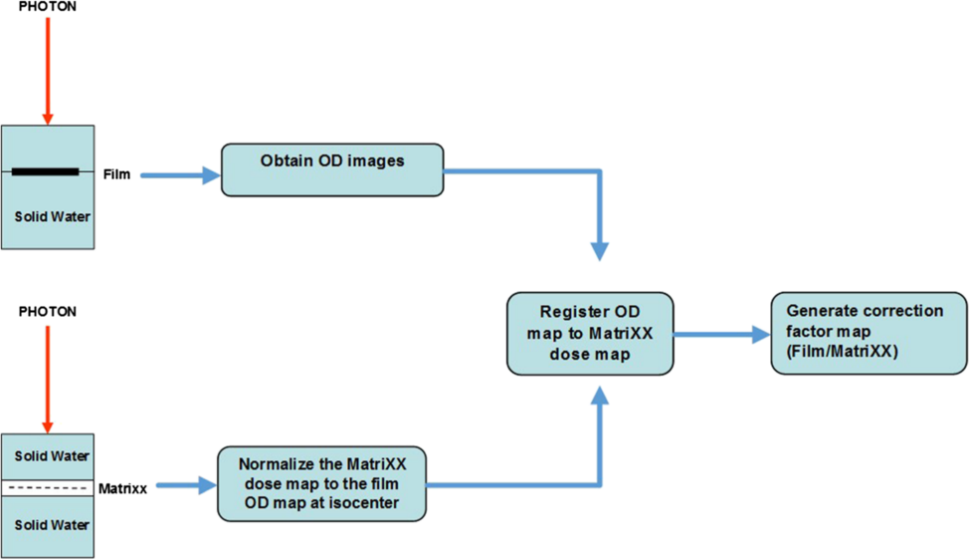
Scanner non-uniform response correction flowchart. The films were irradiated in a solid water phantom at 5 cm depth and the dose distribution was measured with MatriXX at the same setup. The OD images obtained from the films were registered to the normalized MatriXX dose map. The scanner correction map was generated by taking the ratio of the film OD map to MatriXX dose map

A scanner non-uniform response correction is necessary for the following reasons: each charge coupled device detector of a document scanner has different sensitivity, the light source may have non-uniform light intensity, and the amount of scattered light photons also depends on the film location on the scanner bed [11]. It has also been found that the non-uniformity correction depends on the delivered dose [11]. In this study, we designed a three dimensional correction map, which was a function of the scanner bed position and the dose. Figure 2 shows the flowchart of scanner non-uniform response correction. For a given dose level, a solid water phantom (30 × 30 × 15 cm^3^) was setup for irradiation with 5 cm thickness on the top of the film and 10 cm thickness under the film for backscatter. Under the same film irradiation conditions, the reference doses were measured with a 2D ion chamber array (MatriXX, IBA Dosimetry AB, Schwarzenbruck, Germany). The irradiated films were scanned four times and the optical density (OD) maps were averaged from four film OD images. The MatriXX dose map was normalized to the average OD values at the central 1 cm × 1 cm region. The average OD map was registered to the reference MatriXX dose map using an open source image registration tool-kit, Elastix [15]. The pixel-by-pixel correction factors were defined as from the ratio of the OD maps from film to the corresponding dose maps from the diode array. Since the dose map, measured by the ion chamber array, has coarse resolution (7.6 mm pixel-to-pixel distance), it was necessary to resample this map to match the film grid resolution (0.17 mm). In order to minimize the ringing artifact [16], linear interpolation was employed.

### Dose response curve calibration

The mean OD values of central 5 × 5 mm^2^ area were measured on all scanner non-uniformity corrected pre- and post- irradiation. The netOD values were calculated by subtracting the prescan OD values from the corresponding post-scan OD values. The mean netOD and OD values were then fitted to the corresponding reference doses to generate response curves using cubic B-spline interpolation. The sensitivity was calculated for each dose level by taking the first derivative of the dose response curve. Each color channel image of the scanned films was converted to dose using the correction map and dose response curves.

### Film dosimetry uncertainty

Optical density and netOD profiles were evaluated from four film images for each of seven dose levels (1.0, 3.4, 5.3, 7.5, 9.7, 12.0, 15.0 Gy). Film uncertainty, defined as the fluctuation in dose response across the irradiated field, was evaluated by irradiating four films with open fields at various dose levels. All three color channel images from four film images were converted to doses using both netOD-to-dose and OD-to-dose response curves. Pixel–based dose error maps were then calculated from the measured reference doses. Standard deviations were calculated over the central 5 × 5 mm^2^ area for each film between the measured and calculated dose. The root mean square of the standard deviations was calculated over four different films at three dose levels (3.4, 9.7 and 15.0 Gy) for each color channel as a measurement of fluctuation uncertainty.

1 $$ \sigma = \sqrt{\frac{1}{N}\sum_{i=1}^N{\left({x}_i-\overline{x}\right)}^2} $$

where x_i_ is the difference between the measured and calculated dose at pixel i, $\overline{x}$ is the average value of the differences across 5 × 5 mm^2^.

2 $$ {\sigma}_{rms}=\sqrt{\frac{1}{4}\left({\sigma}_1^2+{\sigma}_2^2+{\sigma}_3^2+{\sigma}_4^2\right)} $$

where *σ*_*i*_ is the standard deviation between the measured and calculated dose in the central 5 × 5 mm^2^ area of film i (i = 1 to 4) and *σ*_*rms*_ is the root mean square of the standard deviations.

The time dependency of the films blackening was evaluated using the film calibration dose patterns. EBT3 films from the two different batches were exposed using the same calibration plan (Fig. 1e) and scanned at different time points (0, 0.5, 1.5, 3.5, 7.5 h, 1, 2 and 4 days) to acquire time dependent calibration curves.

### Film dosimetry protocol

#### Film calibration

Figure 1c shows the reference dose measurement conditions. The calibration plans were created for each photon energy in an acrylic phantom (Brainlab, Heimstetten, Germany). The phantom has two 30 × 30 × 5 cm^3^ slabs. The ion chamber plug is drilled right below the surface of the slab, where the distance from the center of plug to the surface is 4.0 mm. Films were positioned in the middle of the phantom with 100 cm SAD setup. Calibration films were irradiated for each photon energy with a nine 2 × 2 cm^2^ square dose pattern ranging from 2.5 to 23.3 Gy (Fig. 1d). The calibration plan was calculated with five 2 × 2 cm^2^ fields shaped by the jaw using the Anisotropic Analytical Algorithm (AAA) dose calculation algorithm (V11.031, Varian Medical Systems, Palo Alto, CA) as shown in Fig. 1e.

The calibration film was scanned, converted to OD image, and registered with the calculated dose plane using a rigid registration based on mutual information. The pixel values of the scanned images were converted to OD images using the following equation:

3 $$ OD=- \log \left({\left[ pv/65535\right]}_{0.1}^{1.0}\right) $$

The variable *pv* represents the pixel values of the scanned image, which was in turn normalized by the maximum pixel value of 65,535. It was then restricted to the range [0.1, 1.0] to prevent the log function from generating very large or infinite numbers.

A binary mask pattern (Fig. 1f) was applied to the registered film image for sampling. The sampled OD values of each color channel were then paired with the calculated dose values to establish the calibration curve utilizing cubic polynomial least squares fitting. The reference doses were also verified with a pin-point 31014 ion chamber (PTW, Freiburg GmbH, Germany).

#### Patient specific quality assurance

Eighty-three SRS/SBRT treatments were performed on a Linac based radiosurgery platform, the Edge, (Varian Medical Systems, Palo Alto, CA) for both intracranial and extracranial diseases. The breakdown of tumor sites treated with SRS/SBRT techniques of the 83 patients in our study were CNS (38 %), lung (24 %), spine (26 %), GI (11 %), and adrenal glands (2 %). The target volume (TV) ranged from 0.04 to 64.36 cc (metastasis) for CNS sites. Median TV volume of SBRT lung patients was 31.59 cc (6.49–116.43 cc). The median TV volume of spine metastases was 40.71 cc (4.72–136.59 cc).

The treatment plans were created in the Eclipse treatment planning system using 6XFFF (1400 MU/min) or 10XFFF mode (2400 MU/min) and the couch model was included in the dose calculation [17]. Treatment techniques used were as follows: 83 % VMAT plans (2.3 ± 0.6 number of arcs), 12 % IMRT plans (8.4 ± 1.7 number of treatment fields), and 5 % Dynamic Conformal Arc plans (3.8 ± 0.5 number of arcs). Dose was delivered in a single fraction (10–18 Gy) for CNS site. All lung cancer patients were treated with 48 Gy in four fractions. All spine metastases patients were treated in a single fraction with the dose ranging from 14 Gy (primary: multiple myeloma) to 18 Gy (all other primary diagnosis).

The same acrylic phantom was used for PSQA. The phantom was setup to measure the dose plane axially for the spine cases to capture dose drop off from the vertical body to the cord and coronally for other cases. For all cases planned with IMRT or VMAT, a single ion chamber measurement (pin point chamber 31014, PTW, Freiburg GmbH, Germany) was made at the isocenter and a film was placed between the slabs to measure planar dose distributions (Fig. 1c). For single isocenter multiple targets (SIMT) cases, both ion chamber and film measurements were made for each target. The percent dose difference ratio between ion chamber measurement and calculation was defined as [(measured dose − calculated dose) × 100 %/calculated dose]. The percentage of points passing gamma based on absolute dose difference was calculated over a region of interest defined at 10 % of maximum dose using 3 %, 1 mm distance-to-agreement criteria. The confidence limit is defined as (│mean│ + 1.96 × standard error) so that 95 % of the data falls within the confidence limit [18]. A one-way ANOVA was used to evaluate if any of the variables (volume size, treatment site, treatment delivery technique) had a statistically significant effect on the point dose measurement and gamma index.

## Results

### Dose response curve calibration

**Fig. 3:**
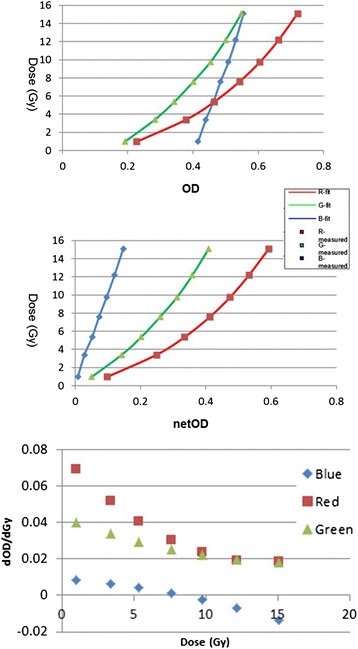
Dose response curves of OD (*upper*) and netOD methods (*middle*) and the sensitivity as a function of dose (*lower*). Cubic B-spline was employed as interpolant (R^2^ = 1 for all the curves)

Figure 3 shows the OD-to-dose (upper) and netOD-to-dose (middle) response curves, as well as the sensitivity as a function of dose (lower). The square dots are the measured OD and netOD values, and the solid lines are the fitted cubic B-spline curves. The red channel sensitivity (defined as OD per unit change in dose) was the highest among all channels in the low dose region, < 10 Gy. The green channel sensitivity was similar to the red channel in the dose range above 10 Gy. The blue channel exhibited the lowest sensitivity in all cases.

### Scanner correction

**Fig. 4:**
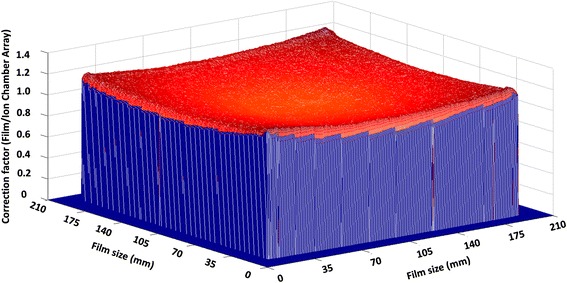
The 3D scanner non-uniform correction map of the 20 × 20 cm^2^ field size at the 12 Gy dose level for the green channel. The *vertical scale* is normalized to 12 Gy

Figure 4 shows a 3D scanner non-uniform correction map of the 20 × 20 cm^2^ field size at the 12 Gy dose level for the green channel. The scanner response varied within 1 % for the field sizes less than 5 × 5 cm^2^, up to 5 % for the field sizes of 10 × 10 cm^2^ and 18 % for the field sizes of 20 × 20 cm^2^ for all color channels.

### Film uncertainty

#### Optical density vs. net optical density

**Fig. 5:**
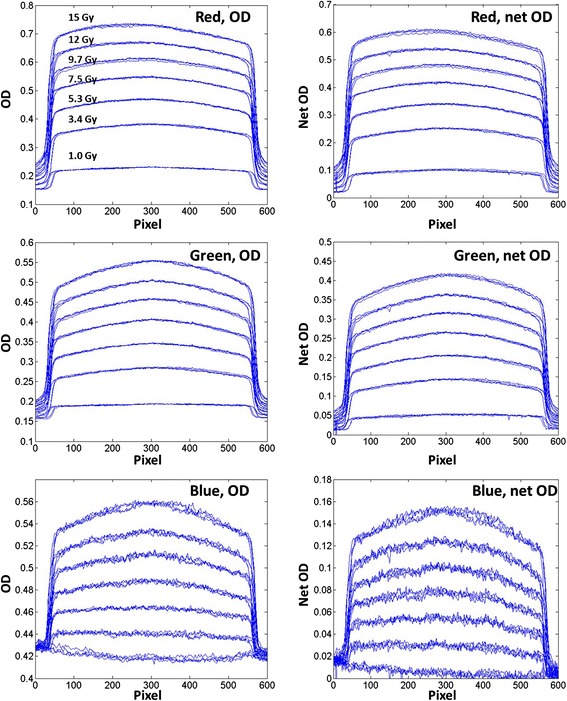
Horizontal (orthogonal to scan direction) OD and netOD profiles for dose levels of 1.0, 3.4, 5.3, 7.5, 9.7 12.0 and 15.0 Gy. The rows from *top* correspond respectively to the red, green, and blue channels. Each *line* represents a profile from one of the four film images

**Fig. 6:**
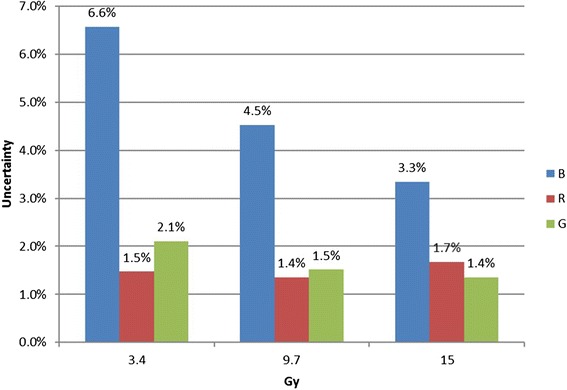
The uncertainty at the three dose levels for the red, green and blue channel

Figure 5 shows the measured OD (left column) and netOD (right column) profiles across the square field centers along the charge-coupled device array direction. The profiles generated from four film images were overlaid at each of the 7 dose levels and evaluated separately for each color channel. The red channel showed a strong lateral dose dependence, which manifested at dose levels above 5.3 Gy. The differences between the OD and netOD variations were minimal. For the green channel, the netOD variations were smaller than that of the red channel, especially at the profile shoulders. The OD response of the blue channel exhibited less fluctuation than that of the netOD response. In addition, at a dose level of 1 Gy, there was little difference between the OD and netOD response curves for the blue channel. Figure 6 shows the uncertainties of the films estimated at three different dose levels for each color channel. The blue channel presented the highest uncertainties for all dose levels, ranging from 3.3 to 6.6 %. For the low dose level at 3.4 Gy, the red channel presented the best accuracy, within 1.5 %. For the higher dose level at 9.7 Gy, the red and green channel had comparable accuracy with an uncertainty level at 1.5 %. For the dose level at 15.0 Gy, a clinically important region for SRS, the uncertainty in the red channel was increased to 1.7 %. The green channel was superior and reached an uncertainty of 1.4 %.

#### Time dependence

**Fig. 7:**
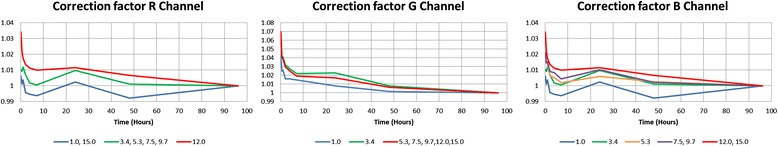
The average correction factors of different dose levels as a function of time, normalized at 4 days after exposure. The starting time of each scanning was recorded in hours

Figure 7 shows the average correction factors for different dose levels as a function of time, normalized at 4 days after exposure. The dose response of the film changed rapidly (~10 %) in the first 2 h (see Fig. 7). The response varied much more gradually after the first 2 h; 3 % change was detected between 2 and 24 h, and another 3 % change occurred between 1 and 4 days. These trends were consistent at the most sensitive regions of each channel, i.e. 3–5 Gy for the red channel, 5–15 Gy for the green channel and 12–15 Gy for the blue channel as shown in Figure 6. For the blue channel, large variation was observed in the correction factor response as a function of time, at a dose level of 1 Gy. The trends were also consistent between two film batches.

### Patient specific quality assurance summary

**Fig. 8:**
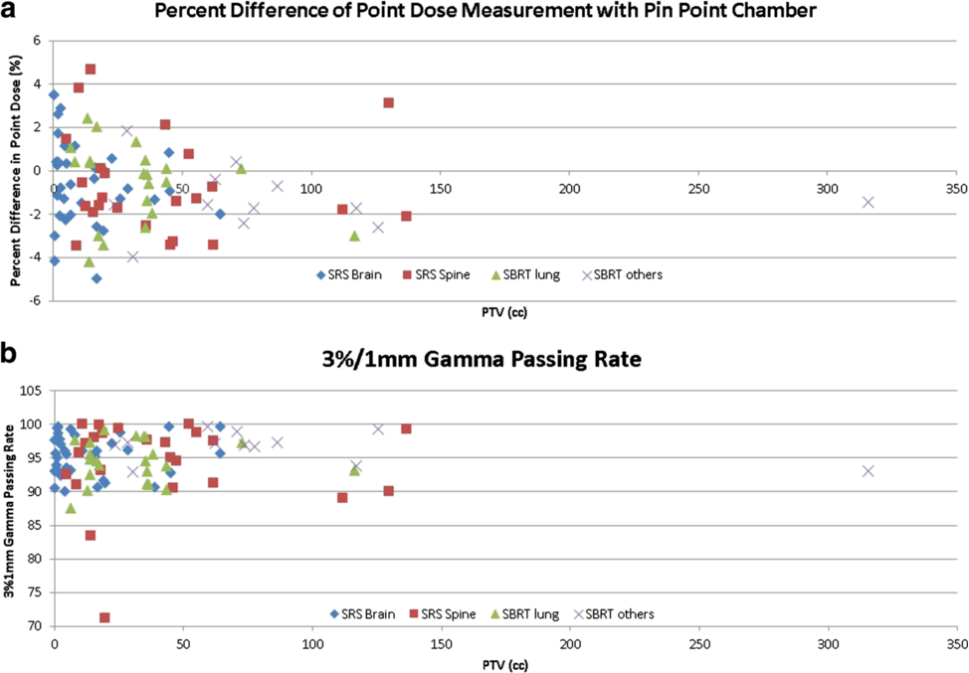
**a** Percent point dose difference between measured (pin point chamber) and calculated dose (Eclipse); **b** The percentage of points passing the gamma criteria of 3 %/1 mm using films based on absolute dose comparison

Figure 8 shows the ion chamber and film measurement results for all plans. The average difference of point dose between measured value using ion chamber and calculated dose from the planning system was −0.64 ± 2.02 for all plans, corresponding to a 95 % confidence interval of (−1.09, −0.26). The percentage of points passing the 3 %/1 mm gamma criteria, averaged over all tests was 95.0 % ± 4.2 %, with a corresponding 95 % confidence interval between 94.2 and 95.9 %.

**Fig. 9:**
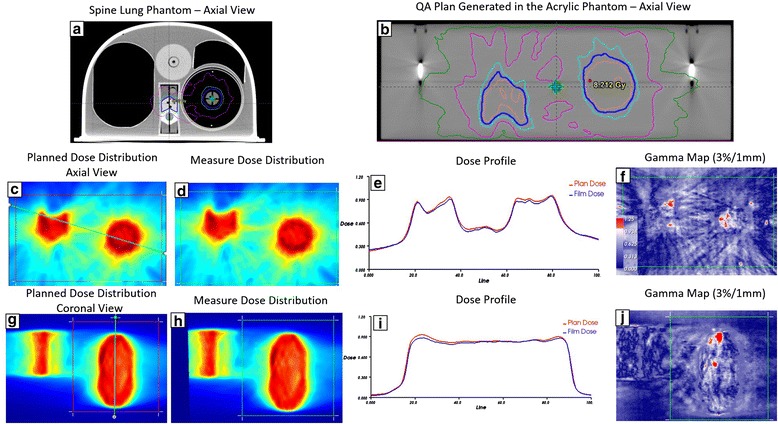
The *upper row*: **a** the spine and lung phantom from IROC for single ISO multiple target irradiation for NRG BR001 and BR002 protocol; **b** Planned axial dose distribution at central level of 6XFFF Hard C-Shape plan using VMAT technique on the acrylic phantom; The *middle row*: (**c**-**d**) Planned and measured dose distribution in the axial plane; **e** The dose profile comparison along the oblique line across both spine and lung targets shown in (**c**); **f** film gamma analysis results with 3 %/1 mm criteria (99.4 % of pixels passed within region of interest shown in (**c**); The *lower row*: The same comparisons in the coronal view. The film gamma pass rate with 3 %/1 mm criteria was 98.9 %

Figure 9 shows an example of film analysis of a QA plan for the spine and lung phantom [Fig. 9 (a)] from the Imaging and Radiation Oncology Core Houston (IROC) for single isocenter multiple target irradiation related to protocols NRG BR001 and BR002. Six Gy was prescribed to both lung and spine target with two arcs using VMAT and 6XFFF following IROC planning guidelines. The QA plan was calculated on the acrylic phantom [Fig. 9 (b)] in both axial and coronal [Fig. 9 (d & h)] orientations. The film placement was intended to replicate the location of the film planes embedded in the spine-lung IROC phantom. The dose difference ratio measured with the pin point chamber was −1.7 %, −2.7 and 0.2 % in the spine, lung and cord region respectively. The line profiles were compared between the calculated and measured dose in the axial plane [Fig. 9 (e)] and the coronal plane [Fig. 9 (i)].

## Discussion

Gafchromic™ film has excellent spatial resolution and is independent of beam angle, energy and dose rate [19]. This makes it suitable for a variety of commissioning and PSQA tasks. However, several sources of uncertainties associated with film dosimetry, such as scanner, background, film uniformity, impact the dosimetric accuracy. Film dosimetry has been used most commonly for relative dose analysis. We systematically investigated the dosimetric uncertainty of EBT3 films. Instead of normalizing the film dose based on ion chamber measurement, we were able to analyze the film based on absolute dose directly. To improve the efficiency of using Gafchromic™ film routinely for patient specific QA, we have developed an integrated film dosimetry protocol that converts OD values to dose, registers film and planar dose and analyzes the profiles and gamma values. The protocol ensures the consistency of film dosimetric results with a clear understanding of the uncertainties of the film dosimetry protocol.

We used the reference doses measured with the MatriXX detector arrays to calculate the scanner pixel-by-pixel non-uniform response correction. The spatial resolution of the MatriXX was limited by the center-to-center distance between the ion chambers. This problem and its potential impact on quality assurance have been investigated by several groups to validate the 2D detector array’s performance [20, 21]. Poppe et al. investigated the sample frequency provided by different 2D arrays and the Nyquist frequency for 1 × 1 cm^2^ field size and complex IMRT fields. They illustrated that the spatial frequency was not extended beyond 0.1 mm^−1^ and very low beyond 0.06 mm^−1^ [4]. Since the spatial frequency of the sampled dose distribution did not exceed one half of the MatriXX sampling frequency 0.085 mm^−1^, i.e. the Nyquist frequency limitation was not violated, the spatial resolution of the MatriXX was appropriate to measure the penumbra region and sharp dose gradient drop off region for small field sizes investigated in this study.

As part of our film dosimetry protocol OD to dose response curve was applied directly. For both the red and green channels, there were negligible differences between the OD and netOD methods, implying that the OD and netOD methods produce the same dosimetric accuracy. However, the profiles from the red channel were skewed due to the scanner lateral dependence artifact at the higher dose levels [22]. For the blue channel, lower doses (e.g. 1 Gy) were insufficient for capturing differences between OD and netOD. Therefore, the images from green channel were extracted and converted to dose using pre-established calibration curves. The dose maps were compared to the treatment planning dose matrix for subsequent profile and gamma analysis for routine SRS/SBRT PSQA.

Film non-uniformity is one of the largest sources of uncertainty for EBT film-based dosimetry. The uncertainties of the Gafchromic^TM^ EBT model have been previously reported in the literature. Battum et al. reported that the uncertainty of 1.8 % was achievable up to a dose range of 2.3 Gy using the red channel when the films were scanned at the central location of the scanner bed [23]. Devic et al. reported 1.5 % uncertainty using the red channel in the dose range of 0~4 Gy [24]. Saur and Frengen reported 2.5 % uncertainty at 2 Gy exposure when scanned in the landscape mode [11] and uncertainty was reduced to 1.7 % when films were scanned in the portrait mode. Papaconstadopoulos et al. suggested a two-color reflection scanning protocol for EBT3 film dosimetry in the dose range of 0 to 8 Gy [25]. Red channel was recommended for doses less than 2 Gy with an accuracy level of 3.7 % and the green channel for higher doses at an accuracy level of 5 %. However, there have been lack of studies to evaluate the uncertainties of EBT3 films for SRS/SBRT treatment. Our study showed that the red channel has very high sensitivity and low uncertainty in the low dose region (<10 Gy) and that the green channel is best suited for most SRS/SBRT prescription dose levels and the overall uncertainty can be controlled at 1.5 %. This uncertainty does not account for the dose response changes of the films as a function of time.

In the time dependency study, the dose difference was greater than 10 % if the film was scanned in the first 2 h after delivery and within 3 % within 1 day thereafter. Calibration curves should be generated at different time periods considering PSQA workflow, such as 4 h for same day delivery and scan, 20 h for the next day scan and 2.5 days if the delivery is done on Friday night and the film is scanned on Monday morning. In our study, four cases did not pass 90 % gamma passing rate, which triggered further evaluation. The passing rate based on absolute dose analysis was 83.4 % (T6 spine), 71.3 % (T9 spine), and 87.6 % (lung, right upper lobe). When the film results were normalized to ion chamber measurements, the corresponding gamma passing rates based on the relative dose analysis were 99.8, 99.7, and 99.6 %. The failure of absolute dose analysis for all the cases was due to the different development time between the calibration and patient films. It is very important to incorporate the impact of time dependent change on OD for absolute dose analysis using Gafchromic^TM^ film.

Our film dosimetry protocol can improve QA efficiency with a single measurement for both point and planar, absolute dose distribution analysis. Our measurements encompassed a variety of complexity in the SRS/SBRT cases. No variables (volume size, treatment site, treatment delivery technique) were found to have a statistically significant impact on the gamma index based on by the one-way ANOVA test. Very limited data have been reported on the SRS/SBRT PSQA using FFF beams [26]. The established confidence limits reported from a large SRS/SBRT patient cohort can be subsequently applied for patient specific verification. The excellent spatial resolution (0.2 mm) of the films, makes it ideal for absolute dose profile analysis, as a means evaluate plan delivery quality for highly modulated dose distributions or small target volumes, as commonly encountered in SRS/SBRT treatments.

## Conclusion

We have estimated the uncertainty of a film dosimetry protocol using Gafchromic™ EBT3 films with a flat-bed document scanner. Utilization of the red channel for low dose range (<10 Gy) and the green channel for high dose range produced the most accurate results in the SRS/SBRT dose regimes. The overall uncertainty of approximately 1.5 % is achievable for the green channel using the EBT3 films if the film delivery and processing is rigorously controlled. The film dosimetry protocol we established offers a highly efficient solution for absolute dose commissioning and routine PSQA for SRS/SBRT treatment procedures.

## Abbreviations

2D: Two-dimensional
AAA: Anisotropic Analytical Algorithm
FFF: Flattening Filter Free
IC: Ionization chamber
IMRT: Intensity modulated radiation therapy
IROC: Imaging and Radiation Oncology Core Houston
MLC: Multi-leaf collimator
OD: Optical density
PSQA: Patient specific quality assurance
SBRT: Stereotactic Body Radiotherapy
SIMT: Single isocenter multiple targets
SRS: Stereotactic Radiosurgery
TIFF: Tagged image file format
TV: Target Volume
VMAT: Volumetric modulated arc therapy
